# Genotypic and Dietary Effects on Egg Quality of Local Chicken Breeds and Their Crosses Fed with Faba Beans

**DOI:** 10.3390/ani11071947

**Published:** 2021-06-29

**Authors:** Tanja Nolte, Simon Jansen, Steffen Weigend, Daniel Moerlein, Ingrid Halle, Henner Simianer, Ahmad Reza Sharifi

**Affiliations:** 1 Department of Animal Sciences, Animal Breeding and Genetics Group, University of Goettingen, 37075 Goettingen, Germany; hsimian@gwdg.de (H.S.); rsharif@gwdg.de (A.R.S.); 2 Center for Integrated Breeding Research, University of Goettingen, 37075 Goettingen, Germany; steffen.weigend@fli.de; 3 Institute of Farm Animal Genetics, Friedrich-Loeffler-Institut, 31535 Neustadt, Germany; simon.jansen@fli.de; 4 Department of Animal Sciences, Division of Quality of Animal Products, University of Goettingen, 37075 Goettingen, Germany; daniel.moerlein@uni-goettingen.de; 5 Institute of Animal Nutrition, Friedrich-Loeffler-Institut, 38116 Braunschweig, Germany; ingrid.halle@fli.de

**Keywords:** egg quality, faba bean, local breeds, vicin

## Abstract

**Simple Summary:**

The quality of chicken eggs is important for reasons of food safety and the consumers’ choice at the point of sale. Faba beans are a regionally produced alternative to soybeans, but they contain substances that could influence the egg quality. The aim of the present study was to test the influence of feeding faba beans on the egg quality of six different chicken genotypes including traditional breeds. The tested chicken genotypes were two local breeds, the Vorwerkhuhn and the Bresse Gauloise, as well as the commercial line White Rock and crossbreds thereof. The genotype had an influence on yolk weight, Haugh units, yolk and shell color, the frequency of inclusions in the eggs and the composition of the eggs. The feeding of faba beans influenced the yolk and shell color as well as Haugh units and shell portion. Egg traits were significantly influenced by the genotype.

**Abstract:**

The quality of chicken eggs is an important criterion for food safety and the consumers’ choice at the point of sale. Several studies have shown that egg quality can be influenced by the chickens’ genotype and by the composition of the diet. The present study aimed to evaluate the effect of faba beans as a substitute for soybeans in the diet of chickens originating from traditional low-performance breeds in comparison with high-performing laying type hens and their crosses on egg quality parameters. Chickens of six different genotypes were fed either with a feed mix containing 20% faba beans with high or low vicin contents or, as a control, a feed mix containing soybeans. The genotypes studied were the local breeds Vorwerkhuhn and Bresse Gauloise, as well as commercial White Rock parent hens and their crosses. Yolk weight, Haugh units, yolk and shell color, the frequency of blood and meat spots and the composition of the eggs were significantly influenced by the genotype. The feeding of faba beans had an effect on yolk and shell color, Haugh units and shell portion, while there was no significant influence on the frequency of blood and meat spots.

## 1. Introduction

Chicken eggs are an important component of human nutrition, because they have a high nutritional value, are cheap to produce and are not subjected to religious restrictions [1]. Since the middle of the last century, poultry production systems have undergone a massive transformation from backyard farming to a highly specialized sector [2,3], which promoted the development and use of genotypes with high laying performance and high egg quality. The utilization of these high-performing lines in commercial poultry production led to the displacement of local chicken breeds due to their comparatively low performance level. Local breeds were since then mainly kept by hobby breeders who ensured their survival but did not systematically select for performance parameters. In the course of the discussion about the killing of day-old male chicks of layer lines, old local breeds came back into the focus of wider interest. Although it is clear that local breeds cannot match the specialized lines regarding performance parameters, economic value and resource efficiency, it is worth evaluating their potential as dual-purpose breeds to supply niche markets and to study how they perform as partners in cross-breeding. However, it is not clear whether the egg quality of local chicken breeds can keep up with that of commercial laying hens.

From the European consumer’s point of view, the most important quality characteristics of eggs are shell strength, albumen consistency and yolk color [4]. The preference for specific yolk colors varies around the world [5], with a darker yellow yolk preferred in Europe [4,6]. The color of the shell is also important to consumers, with different regional preferences. For example, in Europe, brown-shelled eggs dominate, while in the U.S., white-shelled eggs make up the largest part [2]. Furthermore, the albumen consistency, measured as albumen height and converted to Haugh units, serves as an indicator of perceived freshness of eggs [2,4]. Inclusions in the egg, namely blood and meat spots, which develop through the rupture of small blood vessels or displacement of tissue in the oviduct, are generally considered undesirable [7].

It has been shown that these quality parameters are influenced by both the genetics and the composition of the diet. Well known is the effect of different feed components on yolk color, for example, alfalfa, marigold or yellow lupines [8,9]. The egg industry takes advantage of this fact to achieve the right yolk color for certain consumer segments by supplementing feeding stuff additives [10]. Haugh units decrease as the egg ages, but this parameter is also influenced by many other factors, such as genetic background and hen nutrition [5]. In the case of blood spots, Sauter et al. [11] described an influence of nutrition and genetics, but also of the season. The proportions of yolk and albumen are strongly influenced by genetic components. For example, eggs of commercial chicken lines have a higher amount of albumen and less yolk than eggs of local chicken breeds [1,12,13], which is likely due to the selection of commercial lines for higher egg weights and the negative correlation between yolk proportion and egg weight [2]. However, the diet also can influence the shares of yolk and albumen [14,15]. Regarding shell color, Hocking et al. [13] described a high genetic variation within and between commercial lines and traditional breeds. Wilson [16] also described an influence of genetics on shell color variation within lines but pointed out that the diet plays a role, too.

Among the ingredients used in chicken feedstuff, the faba bean (*Vicia faba* L.) is known to affect egg quality. Faba beans contain antinutritional factors, for example, the endogenous glycosides vicin and convicin (together abbreviated as VC). These substances were shown to be responsible for lowered egg and yolk weights, an increasing frequency of blood spots [17], as well as higher values in Haugh units [18].

The objective of the current study was to investigate the influence of feeding faba beans with two different concentrations of VC compared to soybean meal on internal egg quality traits and shell color of two local and one commercial chicken genotype and their crosses. Our focus was to assess whether local breeds with lower egg production levels are better able to compensate for the antinutritive substances contained in the faba bean in terms of egg quality than high-performing genotypes, and whether this makes a difference in their crosses as well.

## 2. Material and Methods

The current experiments were performed in accordance with the German Animal Welfare Law and approved by the Lower Saxony State Office for Consumer Protection and Food Safety (LAVES) (33.19-42502-04-17/2600).

### 2.1. Experimental Design

Two experiments were conducted to evaluate different parameters of egg quality. In experiment A (purebreds), two local chicken breeds and one commercial layer genotype were tested (Table 1). The two local ones were an old German chicken breed, the Vorwerkhuhn (VH), and the French breed Bresse Gauloise (BG). Both breeds have been kept by fancy breeders and were selected according to phenotypic breed standards. While the VH is a layer-type dual-purpose breed from northern Germany, the BG is mainly used for label-meat production in France. The commercial layer hens originated from parent stocks of White Rock (WR) of Lohmann Breeders GmbH (Cuxhaven, Germany). Experiment B (crossbreds) was carried out one year later with the following crosses of the purebreds used in experiment A: Vorwerkhuhn cock × Bresse Gauloise hen (VBG), Vorwerkhuhn cock × White Rock hen (VWR) and Bresse Gauloise cock × White Rock hen (BWR).

Hens were fed three different diets to evaluate the effect on the internal egg quality. The experimental diets contained 20% faba beans (*Vicia faba* L.), either of the vicin-rich variety *Fuego* (VC+) or the vicin-poor variety *Tiffany* (VC−). The VC contents of the diets in experiment A were 0.12% (VC+) and 0.01% (VC−) and 0.13% and 0.02% in experiment B, respectively. The control diet was based on soybean meal (39.8% crude protein; Soy). As further protein source, all diets contained 21% blue sweet lupine (*Lupinus angustifolius cv. Boruta*). The diets were formulated according to GfE (German Society for Nutritional Physiology) recommendations to be isoenergetic and isonitrogenous [19]. A detailed table of ingredients was published before [20] (Supplementary Material Table S1).

The experiments lasted from the 18th until the 52nd week of age. In total, 120 hens per genotype were allocated to six pens of 20 hens each. In combination with the three different diets, this resulted in two replicates of each experimental group (genotype × diet combination). The hens were housed in floor pens equipped with wood chips, perch, dust bath and nine laying nests and had ad libitum access to feed and water. The experimental design and the husbandry conditions were previously described by Nolte et al. [20].

### 2.2. Data Collection

The assessment of internal egg quality was carried out three times for the pure breeds and four times for the crosses during the experiments, at weeks of age 26 (crossbreds only), 34, 42 and 50. Due to an unplanned infestation with the northern fowl mite (*Ornithonyssus sylviarum*) in experiment A, the data obtained in week of age 34 were considered not reliable and therefore excluded from the analysis. As a consequence, only data of week 42 and 50 were used for the purebreds.

On the day before laboratory analysis, 20 eggs of each experimental group (i.e., 10 eggs per pen) were collected randomly. Laboratory analyses started with measuring shell color at two points on the blunt end of the egg with a CM-600d spectrophotometer (Konica Minolta, Munich, Germany). Recorded values were the lightness L*, the redness a* and the yellowness b* of the shell. The blunt end was chosen because it was shown to be representative for the whole egg [21]. Once the eggs were weighed, they were carefully broken on a mirror table. The height of the albumen was measured one centimeter distant from the yolk with the Futura 2a system (Broering information technology, Lohne, Germany), which consists of an albumen height gauge connected to a computer with the appropriate software for data recording. Haugh units were calculated for each egg automatically by the software with the formula $HU=100*log\left(h-1.7w^{0.37}+7.6\right)$, where h is the albumen height and w is the egg weight. Placed on the mirror table, the broken eggs were visually examined for blood and meat spots. Blood spots were defined as located at the yolk, while meat spots were found in the albumen [7]. Yolk and albumen were separated from each other and the remains of the albumen on the yolk were removed by rolling the yolk carefully on a paper tissue. The yolk was weighed and the color determined with the Roche color fan (DSM nutritional products GmbH, Grenzach, Germany).

Albumen weight was calculated by subtracting the yolk and shell weight from the egg weight, whereas shell weights of all eggs analyzed in this study were available from the parallel analysis of external egg quality parameters [20]. Relative proportions of the various egg components such as yolk, albumen and shell were calculated from the quotient between the respective weight and egg weight.

### 2.3. Statistical Analysis

The data were analyzed with linear mixed models using the ‘GLIMMIX’ procedure of the statistical program SAS (SAS 9.3, SAS institute Inc., Cary, NC, USA). The two experiments (purebreds, crossbreds) were analyzed separately. The statistical model for the analysis of yolk weight, yolk color, Haugh units, yolk, albumen and shell percentage was as follows:

(1) $$Y_{ijklm}=\mu +G_{i}+D_{j}+A_{k}+G_{i}D_{j}+G_{i}A_{k}+D_{j}A_{k}+G_{i}D_{j}A_{k}+p_{l}+e_{ijklm}$$

where $Y_{ijklm}$ is the respective trait variable, μ is the overall mean, $G_{i}$ is the fixed effect of genotype, $D_{j}$ is the fixed effect of diet, $A_{k}$ is the fixed effect of age, $G_{i}D_{j}$, $G_{i}A_{k}$, $D_{j}A_{k}$, $G_{i}D_{j}A_{k}$ are the interactions of the respective factors, $p_{l}$ is the random effect of the pen and $e_{ijklm}$ is the random error. The values of yolk, albumen and shell percentage were subjected to an arcsine transformation before analysis. The presented least squares means (LS-means) were then back-transformed to percentages. A similar statistical model was used for the analysis of shell color (L*, a* and b* values), whereby the repeated measurements on the individual egg $\left(I\right)$ were considered as random effect in the model as follows:

(2) $$Y_{ijklm}=\mu +G_{i}+D_{j}+A_{k}+G_{i}D_{j}+G_{i}A_{k}+D_{j}A_{k}+G_{i}D_{j}A_{k}+p{\left(I\right)}_{l}+e_{ijklm}$$

The number of blood and meat spots was analyzed by applying a linear logistic model as follows:

(3) $$log\left(\frac{\pi_{ijk}}{1-\pi_{ijk}}\right)=\varphi +G_{i}+D_{j}+A_{k}+G_{i}D_{j}+G_{i}A_{k}+D_{j}A_{k}+G_{i}D_{j}A_{k}$$

where $\pi_{ijk}$ is the probability for the occurrence of blood or meat spots, φ is the overall mean, $G_{i}$ is the fixed effect of genotype, $D_{j}$ is the fixed effect of diet and $A_{k}$ is the fixed effect of age, $G_{i}D_{j}$, $G_{i}A_{k}$, $D_{j}A_{k}$, $G_{i}D_{j}A_{k}$ are the interactions of the respective factors. LS-means were estimated on the logit scale and then back-transformed to the original scale (probability) by using the inverse link function [22].

For all parameters, significant differences between least squares means were tested using a *t*-test procedure by inclusion of the PDIFF option in the LSMEANS statement (SAS, 2018).

## 3. Results

The effects of genotype, diet, age and their interactions on the different egg quality parameters of the purebred hens are presented in Table 2.

**Figure 1:**
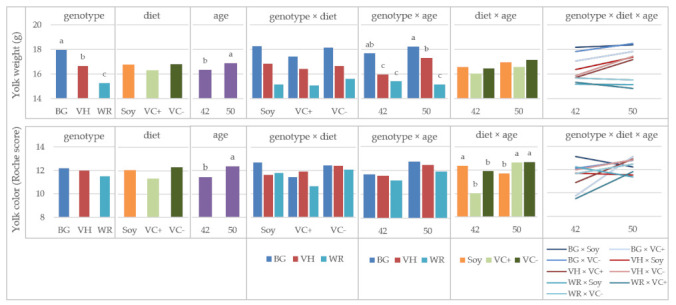
Yolk weight and color in purebred chicken, LS-means for the effects of genotype, diet, age and their interactions; BG: Bresse Gauloise, VH: Vorwerkhuhn, WR: White Rock; ^a,b,c^ Bars in one diagram not sharing a letter differ at *p* < 0.05, letter codes only shown for significant effects.

Yolk weight was influenced by genotype, age and their interaction. The BG showed that the highest yolk weight (17.96 g), the weights of VH (16.66 g) and WR (15.29 g) were significantly lower (Figure 1). Regarding the effect of age, the yolk weight significantly increased from week 42 to 50 by 0.55 g. In contrast to the local breeds, the yolk weight of WR decreased with increasing age.

Yolk color was not influenced by the main factors genotype and diet, but by the age, the interaction of diet and age and the threefold interaction of all factors. There was an increase in yolk color score from week 42 to 50 by one tint of the Roche color fan (Figure 1). This effect could actually be seen in the VC+ and VC− groups of all genotypes, although not statistically significant for VC−. On the contrary, in the soy groups a brightening of yolk color with aging was observed. Comparing the feeding groups between both measurements, the changes were statistically significant in all diets.

**Figure 2:**
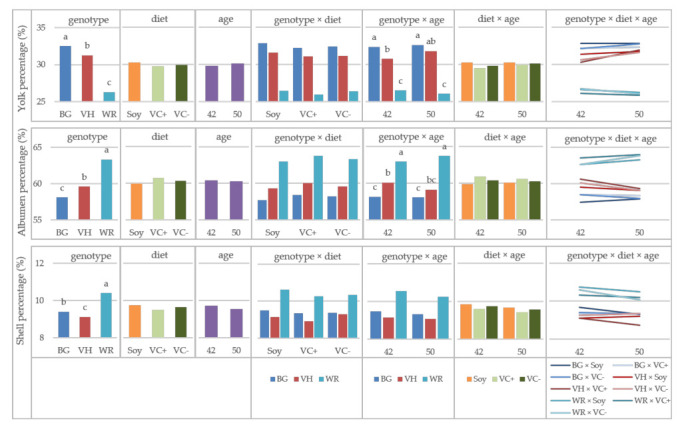
Egg components in purebred chicken, LS-means for the effects of genotype, diet, age and their interactions; BG: Bresse Gauloise, VH: Vorwerkhuhn, WR: White Rock; ^a,b,c^ Bars in one diagram not sharing a letter differ at *p* < 0.05, letter codes only shown for significant effects.

All egg components were significantly influenced by the main factor genotype. For yolk and albumen percentages as well interactions of genotype × age were observed (Table 2). The yolk percentage was highest in the local breeds BG (32.49%) and VH (31.26%), whereas the WR yolk amounted to 26.28%. All genotypes differed significantly from each other (Figure 2).

While the portion of yolk in the local breeds increased with aging of the hens, the WR showed a decrease. However, these changes were only small and not statistically significant. The albumen percentage was highest in the WR (63.30%) and lowest in BG (58.08%). The interaction of genotype and age corresponded to a decrease of albumen percentage from week 42 to 50 in BG and VH, while it increased in WR, as well only minimal and not statistically significant. The shell portion was highest in WR (10.41%) and significantly lower in BG (9.40%) and VH (9.12%).

Eggshell lightness (L*) was influenced by the main factors genotype and diet and their interaction as well as by the age (Table 2). Only in WR chicken was a significant difference between feeding groups observed (Table 3), meaning that the VC− groups produced eggs with a slightly lower shell lightness than the Soy and VC+ groups. With regard to the difference between genotypes, the shell color of BG and VH was cream, while the WR laid dark brown eggs. Therefore, the BG and VH showed significantly higher L* values than the genotype WR. With aging of the hens, the lightness of the eggshell increased significantly from 74.42 to 76.31. The redness (shell a*) was influenced by the genotype and age of the hens. All genotypes differed significantly, with WR having the highest a* value and VH the lowest. A significant decrease in a* value was recorded with increasing age. The yellowness, expressed as b* value, behaved similar to the redness. A significant influence of the diet was observed. This is, however, not reflected in the LS-means, as the feeding groups do not differ significantly from each other.

For Haugh units, two main factors, i.e., genotype and diet, were identified by ANOVA. All genotypes differed significantly from each other, with WR showing the highest values and VH the lowest values (Table 4). With regard to the feed treatment, in the VC+ groups significantly higher Haugh units have been measured than in the Soy and VC− groups.

Only the genotype had a significant influence on the frequency of blood spots (Table 2). The WR showed blood spots in more than half of the eggs examined. The frequency in VH and BG was significantly lower. The incidence of meat spots was neither influenced by genotype, age or diet, nor were there any significant interactions between factors.

**Figure 3:**
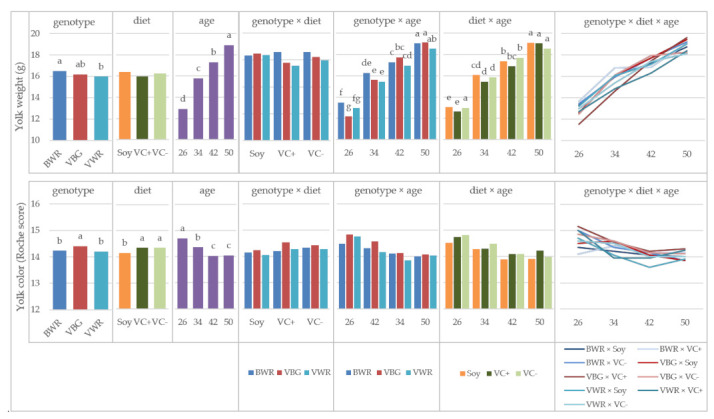
Yolk weight and color in crossbred chicken, LS-means for the effects of genotype, diet, age and their interactions; VBG: VH male × BG female, VWR: VH male × WR female, BWR: BG male × WR female; ^a,b,c,d,e,f,g^ Bars in one diagram not sharing a letter differ at *p* < 0.05, letter codes only shown for significant effects.

Regarding crossbreed chickens in experiment 2, the effect of genotype, diet, age and their interactions on egg quality parameters is displayed in Table 5. Yolk weight was influenced by the main factor genotype, with BWR showing significantly heavier yolks than VWR (16.51 g vs. 15.97 g; Figure 3). As well, age had a significant influence on yolk weight, which was expressed in increasing yolk weights with aging. Between age and the main factors, interactions existed. Figure 3 shows that the increase of yolk weight is different between genotypes: VBG showed the highest gain of 6.89 g during the experiment, whereas the weight gain in BWRs yolks was only 5.54 g. Furthermore, in week 26, there was a significant difference between these two genotypes. The interaction of diet and age shows differences in the increase of yolk weight between feeding groups. The highest increase was observed for VC+ (6.38 g) and the lowest for VC− (5.56 g).

**Figure 4:**
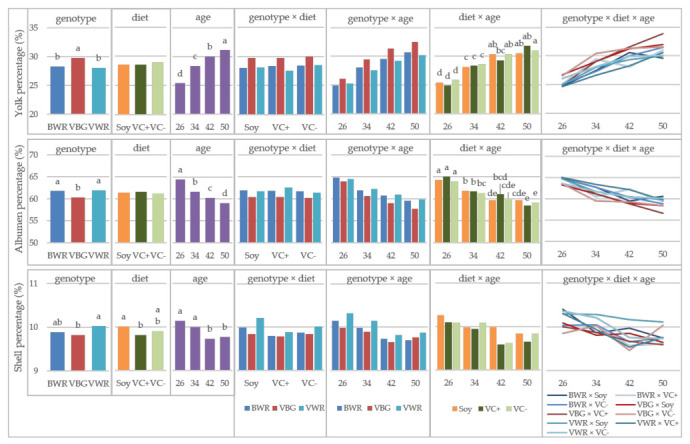
Egg components in crossbred chicken, LS-means for the effects of genotype, diet, age and their interactions; VBG: VH male × BG female, VWR: VH male × WR female, BWR: BG male × WR female; ^a,b,c,d,e^ Bars in one diagram not sharing a letter differ at *p* < 0.05, letter codes only shown for significant effects.

Yolk color of the crossbreds was influenced by the effects of genotype, diet, age and their three-way interaction. During the experiment, a brightening of yolk color took place in almost all experimental groups with increasing age (Figure 3). The highest difference was shown of the BWR VC−group of 1.2 nuances of the Roche color score. In contrast, the difference in the BWR Soy group was only −0.3 and in the VC+ group +0.1. Looking at the single effects of the main factors the VBG showed significantly darker yolks than BWR and VWR, but it must be mentioned that this difference was less than 0.2 Roche tones. The same is true for the effect of diet, where the soy groups show statistically significant brighter yolks than the faba bean groups. In Figure 4, the egg components of the crossbreds are shown. Yolk and albumen percentage were significantly influenced by the genotype, the age, the interaction of diet × age and the three-way interaction of all three factors. The VBG had the significantly highest portion of yolk of 29.80%, whereas the WR crosses achieved 28.78% (BWR) and 28.03% (VWR), respectively. A statistically significant increase of yolk percentage with increasing age amounting in total to 5.71% could be observed. The general trend of increasing yolk percentage was not true for all experimental groups, indicated by significant interactions between the three factors genotype, diet and age. In the BWR Soy group from week 42 to 50, a decrease of almost 1% took place. Furthermore, in the BWR VC+ group, a decrease from 29.43% to 28.11% from week 34 to week 42 was observed, which was compensated by an increase of up to 31.01% measured in week 50. In the case of albumen percentage, the effects of genotype and age behaved exactly the other way round. The VWR and BWR showed significantly higher portions than the VBG (61.91% and 61.80% vs. 60.34%). With aging, the albumen percentage was lowered by 5.35% over the experimental period. As well, in this parameter, the BWR groups behaved differently than the general trend. The BWR Soy group was characterized by an increase of the albumen percentage from week 42 to 50, whereas the increase in the BWR VC+ group took place from week 34 to 42, followed again from a decrease of albumen percentage towards week 50. The shell percentage was relevantly influenced by genotype, diet and age. The VWR revealed a significantly higher portion of shell than the VBG (10.03% vs. 9.82%), the BWR being intermediate (9.89%). Of the feeding groups, the Soy group showed a 0.19% higher portion of shell than the VC+ group. With respect to the age, there was a statistically significant decrease of shell portion observed from week 34 to week 42.

All parameters of eggshell color (lightness L*, redness a* and yellowness b*) were significantly affected by genotype, diet and age (Table 5). The VBG showed significantly higher lightness and significantly lower a* and b* values than VWR and BWR, which did not differ significantly from each other (Table 6). Regarding the dietary effect, the VC+ group revealed significantly higher L* and significantly lower a* and b* values than the VC− group. The Soy group behaved intermediately. The effect of age was different between the parameters. In case of the L* value, in week 34 and 50, the value was significantly lower than in week 26. The redness was significantly higher in week 34 than in week 50. The b* was significantly lower in week 26 than in weeks 34 and 42, which did not differ. In week 50, the b* value was significantly lower than at all other time points.

The Haugh units for the crossbred chickens are displayed in Table 7. The main factors, genotype, diet and age, significantly influenced the Haugh units (HU) as well as the interaction of genotype × diet. In VBG, the VC+ group showed significantly higher HU than the VC− group, while in BWR the VC+ group achieved significantly higher HU than the Soy group. In the main effect of genotype, BWR showed the highest HU followed by VWR and VBG, all differing significantly from each other. With respect to the diet, the VC+ groups had significantly higher HU than Soy and VC−. Aging of hens led to a decrease of HU from week 26 to 34 and to 42, with weeks 42 and 50 not differing statistically significantly.

Bloodspots differed between genotypes, with BWR showing the highest frequency and VBG the lowest. The frequency of meat spots was not significantly influenced by any of the tested effects.

## 4. Discussion

In purebred chicken, the yolk weight of the local breeds BG and VH was higher than that of the commercial line WR. A similar difference between local and commercial chickens was also described in several studies comparing different commercial lines and local breeds [1,12,13]. Moreover, Rizzi and Chiericato [23] observed that increasing age of hens led to an increase in yolk weight of Italian local breeds but not in commercial hybrids. The same was shown in the present study. Regarding the diet, some authors described VC leading to lowered yolk weights [17,24]. This cannot be confirmed by the present study.

Concerning the yolk color, a remarkable increase in color score was observed from week 42 to 50 in the VC+ groups of all genotypes. As noted above, there was an infestation of the Northern Fowl mite in the stock around week 34 that led to severe performance losses mainly in the VC+ groups. Both the feeding of faba beans and an infection with fowl mites challenge the immune system [25,26,27] and influence therefore metabolic processes in the liver. Given that yolk pigments are partly built in the liver, a causal connection between the previous exposure to metabolic stress and the relatively bright yolk colors of the VC+ groups in week 42 might be possible. However, this observation was found by chance, and a more detailed investigation of such a relationship requires further research with a specific experimental design. The increase in color score of the VC− groups was much weaker with less than 1 Roche nuance from weeks 42 to 50, while on the other hand, the Soy groups showed a light brightening. In literature, both darker and brighter yolk color under the feeding of faba beans was described [28,29], as well as no effect [18].

Egg components showed genotypic differences as expected with the local breeds’ eggs having a higher portion of yolk and less albumen and shell percentages than the commercial line WR. The breeding for higher egg weights led to a relative increase of albumen, and the breeding for high shell stability led to a higher portion of shell. The genotype × age interaction demonstrated that the genotype differences in yolk and albumen portion become even more clear with aging [23].

Shell color is determined genetically and therefore differs between genotypes. The WR is a brown layer line, while the egg shell colors of BG and VH are described as white or yellowish [30,31]. Although the effects of diet, genotype × diet and age as well have been statistically significant in the analysis, these caused only tiny changes that were scarcely visible nuances to the human eye. However, an influence of feed on eggshell lightness (L*) was recently reported by Mori et al. [32], comparing mixed and fermented feed.

The dependence of Haugh units on chicken genotype is controversially discussed in the literature. Haugh units of local chickens have been higher [6], lower [13] or in between [1] that of commercial lines. The genotype differences were confirmed in the present study, with the commercial genotype showing the highest values. The effect of faba beans on Haugh units is also not distinct. In our study, the highest Haugh units were observed in the groups fed with the VC+ diet. While in some studies an increase of Haugh units along with increased faba bean levels was observed [28,33], Lessire et al. [18] ascribed this effect to VC, leading to higher viscosity of the albumen. In contrast, Daenner [34] did not observe a change in Haugh units while feeding different levels of vicin-rich and vicin-poor faba beans.

In the present study, WR showed a much higher frequency of blood spots than the local genotypes, while in the case of meat spots, the differences between the genotypes were not significant. Hocking et al. [13] found no difference between the frequency of blood spots in traditional breeds compared to commercial lines and, similarly, Sauter et al. [11] negated an influence of laying performance on the amount of blood spots. Brade et al. [7] stated that brown-shelled eggs in general have more blood and meat spots compared to white-shelled eggs. No influence of faba bean feeding on the frequency of blood and meat spots was detected in the present study. This is in accordance with the results of Lessire et al. [18] but contradictory to Muduuli et al. [17], who described four times more blood spots in eggs of hens that were fed 1% vicin in the diet compared to the control group. Robblee et al. [33] also observed a slight increase in the number of blood spots.

Yolk weight of the crossbreds was influenced by the three-way interaction of all factors. There was a trend towards increased yolk weights as hens aged, although its magnitude differed between genotypes and feeding groups. No clear direction of the interaction is visible.

Although statistically significant differences in yolk color were detected, these are of minor relevance, as they were less than one nuance on the Roche color fan.

In general, there is a trend of increasing yolk and decreasing albumen portion with aging of the hens, which was also observed in the local breeds in Experiment A. For the effect of crossbreeding a local with a commercial genotype, this could be a favorable effect, as yolk is the part of the egg containing the valuable ingredients [35].

The shell color tones of the crossbreds’ eggs were mixtures of the colors of the parental lines, i.e., light brown in the case of VWR and BWR and white to tinted in VBG. Li et al. [21] observed a similar effect with the crosses of white and brown layers and suggested additive effects of the genes responsible for eggshell color resulting in a mixture of color. Similar as in experiment A, the significant differences in L*, a* and b* values between feeding groups and measurements are negligible, because they were not visible with the human eye at all.

The genotype × diet interaction in Haugh units showed different responses of the crossbreds towards the diets. As described above, the information from other studies regarding the effect of faba beans on Haugh units was not the same between experiments. This is possibly due to the different commercial genotypes used in the respective experiments.

Our results suggest that the genetic predisposition to blood spots was transferred from WR hens to their crossbred offspring. While the frequency in BWR was slightly lower compared to the parental WR, the crossbreeding of WR with VH reduced the frequency of blood spots by half. This reduction was also observed when the local breeds were crossed with each other (VBG).

## 5. Conclusions

All crossbred genotypes, especially the two crosses with WR hens, revealed an internal egg quality that is comparable to that of commercial layers.

The apparent susceptibility of WR hens to blood spots is significantly reduced in the progeny of these birds when crossed with the local breeds.

In our companion publication addressing the egg production traits and bone stability of these hens, we concluded BWR to be the most promising genotype of the evaluated crossbreds regarding dual-purpose use [20]. This is still true, although the BWR genotype has the disadvantage of a high frequency of blood spots in the eggs, even though it is lower than WR.

Assembling the present study with our previous publications regarding the egg production traits and bone stability of the hens [20] and the fattening performance of the male counterparts [36], it becomes again apparent that faba beans at the portion of 20% are a suitable alternative to soybeans at least for the investigated genotypes.

## Figures and Tables

**Table 1:** Experimental design.

	Experiment A	Experiment B
genotypes	Bresse Gauloise (BG)	BG cock × WR hen (BWR)
	Vorwerkhuhn (VH)	VH cock × BG hen (VBG)
	White Rock (WR)	VH cock × WR hen (VWR
diets	Control diet based on soybean meal (Soy)	
	20% vicin-rich faba bean (*Fuego*; VC+)	
	20% vicin-poor faba bean (*Tiffany*; VC−)	
number of birds	120 per genotype	
replicates	2	

**Table 2:** The effect of genotype, diet, age and their interactions on parameters of internal egg quality in purebred chicken.

Parameter	Genotype	Diet	Age	Genotype × Diet	Genotype × Age	Diet × Age	Genotype × Diet × Age
Yolk weight	**<0.0001**	0.0844	**0.0001**	0.6404	**<0.0001**	0.7032	0.7622
Yolk color	0.2420	0.0525	**<0.0001**	0.5153	0.5724	**<0.0001**	**0.0221**
Yolk percentage	**<0.0001**	0.3713	0.2232	0.9889	**0.0191**	0.6482	0.7768
Shell percentage	**<0.0001**	0.0789	0.0540	0.4343	0.6163	0.9988	0.3795
Albumen percentage	**<0.0001**	0.0739	0.6854	0.9959	**0.0081**	0.6494	0.7859
Shell color L*	**<0.0001**	**0.0110**	**<0.0001**	**0.0451**	0.9935	0.2120	0.7545
Shell color a*	**<0.0001**	0.0561	**<0.0001**	0.1267	0.1554	0.7685	0.5547
Shell color b*	**<0.0001**	**0.0457**	**0.0031**	0.4040	0.0571	0.1707	0.8498
Haugh units	**<0.0001**	**<0.0001**	0.5972	0.2412	0.5441	0.8835	0.5985
Blood spots	**0.0009**	0.3186	0.8136	0.9479	0.5911	0.5676	0.1443
Meat spots	0.7576	0.9002	0.9534	0.8437	0.1812	0.9006	0.5985

*p*-values, significant results (*p* < 0.05) are accentuated in bold numbers.

**Table 3:** Least-squares means ± SE for the effect of genotype, diet, age and the genotype × diet interaction in purebred groups on shell color (L*, a* and b* values).

Effect	Shell L*	Shell a*	Shell b*
Genotype			
BG	84.55 ± 0.32 ^a^	3.26 ± 0.17 ^b^	13.78 ± 0.31 ^b^
VH	85.05 ± 0.32 ^a^	2.60 ± 0.17 ^c^	12.50± 0.31 ^c^
WR	56.50 ± 0.32 ^b^	19.82 ± 0.17 ^a^	29.25 ± 0.31 ^a^
Diet			
Soy	76.01 ± 0.32 ^a^	8.46 ± 0.17	18.80 ± 0.31
VC+	75.44 ± 0.32 ^ab^	8.33 ± 0.17	17.88 ± 0.31
VC−	74.66 ± 0.32 ^b^	8.89 ± 0.17	18.86 ± 0.31
Age (weeks)			
42	74.42 ± 0.26 ^b^	8.97 ± 0.14 ^a^	19.05 ± 0.25 ^a^
50	76.31 ± 0.26 ^a^	8.15 ± 0.14 ^b^	17.97 ± 0.25 ^b^
Genotype × Diet			
BG × Soy	84.69 ± 0.55 ^a^	3.32 ± 0.30	13.75 ± 0.54
BG × VC+	84.14 ± 0.55 ^a^	3.32 ± 0.30	13.64 ± 0.54
BG × VC−	84.81 ± 0.55 ^a^	3.14 ± 0.30	13.95 ± 0.54
VH × Soy	85.47 ± 0.55 ^a^	2.64 ± 0.30	12.76 ± 0.54
VH × VC+	85.80 ± 0.55 ^a^	2.01 ± 0.30	11.37 ± 0.54
VH × VC−	83.89 ± 0.55 ^a^	3.16 ± 0.30	13.39 ± 0.54
WR × Soy	57.86 ± 0.55 ^b^	19.44 ± 0.30	29.89 ± 0.54
WR × VC+	56.37 ± 0.55 ^bc^	19.64 ± 0.30	28.62 ± 0.54
WR × VC−	55.27 ± 0.55 ^c^	20.37 ± 0.30	29.24 ± 0.54

BG: Bresse Gauloise, VH: Vorwerkhuhn, WR: White Rock; ^a,b,c^ Values in one column and effect not sharing a letter differ significantly at *p* < 0.05.

**Table 4:** Least-squares means ± SE for the effect of genotype and diet in purebred groups on Haugh units, blood and meat spots.

Effect	Haugh Units	Blood Spots (%)	Meat Spots (%)
Genotype			
BG	74.71 ± 0.66 ^b^	15.28 ± 3.85 ^a^	13.15 ± 3.51
VH	67.56 ± 0.66 ^c^	7.69 ± 2.71 ^a^	16.94 ± 3.74
WR	87.37 ± 0.66 ^a^	52.59 ± 5.21 ^b^	3.21 ± 168.30
Diet			
Soy	75.04 ± 0.66 ^b^	23.51 ± 5.18	2.10 ± 111.57
VC+	78.97 ± 0.66 ^a^	24.62 ± 5.12	16.68 ± 3.73
VC−	75.61 ± 0.66 ^b^	14.24 ± 4.22	19.23 ± 4.15

BG: Bresse Gauloise, VH: Vorwerkhuhn, WR: White Rock; ^a,b,c^ Values in one column and effect not sharing a letter differ significantly at *p* < 0.05.

**Table 5:** The effect of genotype, diet, age and their interactions on parameters of internal egg quality in crossbred chicken.

Parameter	Genotype	Diet	Age	Genotype × Diet	Genotype × Age	Diet × Age	Genotype × Diet × Age
Yolk weight	**0.0088**	0.0723	**<0.0001**	0.1216	**<0.0001**	**0.0168**	**0.0099**
Yolk color	**0.0135**	**0.0128**	**<0.0001**	0.6096	0.0984	0.4923	**0.0465**
Yolk percentage	**<0.0001**	0.4763	**<0.0001**	0.7766	0.1651	**0.0002**	**<0.0001**
Albumen percentage	**<0.0001**	0.4129	**<0.0001**	0.7064	0.1161	**0.0004**	**<0.0001**
Shell percentage	**0.0056**	**0.0139**	**<0.0001**	0.5859	0.9079	0.1994	0.5292
Shell color L*	**<0.0001**	**0.0109**	**0.0085**	0.2232	0.1173	0.7424	0.2797
Shell color a*	**<0.0001**	**0.0212**	**0.0058**	0.2614	0.2981	0.2900	0.1993
Shell color b*	**<0.0001**	**0.0099**	**<0.0001**	0.5107	0.8808	0.7680	0.2584
Haugh units	**<0.0001**	**0.0040**	**<0.0001**	**0.0089**	0.2369	0.2822	0.5560
Blood spots	**<0.0001**	0.7914	0.4928	0.8743	0.3978	0.8752	0.6308
Meat spots	0.9996	1.0000	1.0000	1.0000	0.9621	0.7880	0.9684

*p*-values, significant results (*p* < 0.05) are accentuated in bold numbers.

**Table 6:** Least-square means ±SE for the effect of genotype, diet and age in crossbred groups on shell color (L*, a* and b* values).

Effect	Shell L*	Shell a*	Shell b*
Genotype			
VBG	81.85 ± 0.27 ^a^	5.05 ± 0.16 ^b^	16.78 ± 0.21 ^b^
VWR	68.67 ± 0.27 ^b^	13.69 ± 0.16 ^a^	25.40 ± 0.21 ^a^
BWR	67.98 ± 0.27 ^b^	13.82 ± 0.16 ^a^	25.90 ± 0.21 ^a^
Diet			
Soy	72.79 ± 0.27 ^ab^	10.89 ± 0.16 ^ab^	22.77 ± 0.21 ^ab^
VC+	73.42 ± 0.27 ^a^	10.51 ± 0.16 ^b^	22.21 ± 0.21 ^b^
VC−	72.29 ± 0.27 ^b^	11.15 ± 0.16 ^a^	23.09 ± 0.21 ^a^
Age (weeks)			
26	73.70 ± 0.31 ^a^	10.61 ± 0.19 ^ab^	22.44 ± 0.24 ^b^
34	72.30 ± 0.31 ^b^	11.26 ± 0.19 ^a^	23.48 ± 0.24 ^a^
42	72.80 ± 0.31 ^ab^	11.09 ± 0.19 ^ab^	23.65 ± 0.24 ^a^
50	72.54 ± 0.31 ^b^	10.45 ± 0.19 ^b^	21.20 ± 0.24 ^c^

VBG: Vorwerkhuhn male × Bresse Gauloise female, VWR: Vorwerkhuhn male × White Rock female, BWR: Bresse Gauloise male × White Rock female; ^a,b,c^ Values within one column and effect not sharing a letter differ significantly at *p* < 0.05.

**Table 7:** Least-square means ± SE for the effect of genotype and diet in crossbred groups on Haugh units and egg inclusions.

Effect	Haugh Units	Blood Spots (%)	Meat Spots (%)
Genotype			
VBG	77.23 ± 0.60 ^c^	3.72 ± 97.13	11.70 ± 2.23
VWR	81.01 ± 0.60 ^b^	24.06 ± 2.76 ^a^	1.00 ± 103.16
BWR	85.12 ± 0.60 ^a^	44.46 ± 3.53 ^b^	3.67 ± 260.50
Diet			
Soy	80.42 ± 0.60 ^b^	24.31 ± 3.06	3.16 ± 225.71
VC+	82.75 ± 0.60 ^a^	21.24 ± 3.28	3.44 ± 244.65
VC−	80.19 ± 0.60 ^b^	10.16 ± 247.56	4.20 ± 296.63
Age (weeks)			
26	85.56 ± 0.69 ^a^	20.15 ± 3.82	3.54 ± 335.59
34	81.84 ± 0.69 ^b^	27.82 ± 3.97	1.94 ± 186.71
42	78.76 ± 0.69 ^c^	7.40 ± 247.70	7.88 ± 1.88
50	78.32 ± 0.70 ^c^	21.24 ± 3.48	2.95 ± 281.82
Genotype × Diet			
VBG × Soy	76.63 ± 1.04 ^de^	11.44 ± 3.84	10.41 ± 3.56
VBG × VC+	79.84 ± 1.04 ^cd^	8.56 ± 3.41	11.99 ± 4.02
VBG × VC−	75.22 ± 1.04 ^e^	0.47 ± 38.41	12.82 ± 4.12
VWR × Soy	81.92 ± 1.04 ^bc^	26.95 ± 5.00	0.32 ± 70.30
VWR × VC+	80.40 ± 1.04 ^bcd^	21.24 ± 4.79	0.28 ± 62.66
VWR × VC−	80.70 ± 1.04 ^bcd^	24.21 ± 4.82	10.14 ± 3.50
BWR × Soy	82.71 ± 1.04 ^bc^	41.01 ± 5.64	8.56 ± 3.54
BWR × VC+	88.01 ± 1.04 ^a^	43.71 ± 5.89	10.41 ± 3.59
BWR × VC−	84.64 ± 1.04 ^ab^	48.72 ±5.83	0.50 ± 111.10

VBG: Vorwerkhuhn male × Bresse Gauloise female, VWR: Vorwerkhuhn male × White Rock female, BWR: Bresse Gauloise male × White Rock female; ^a,b,c,d,e^ Values within one column and effect not sharing a letter differ significantly at *p* < 0.05.

## Data Availability

The data presented in this study are available on reasonable request from the corresponding author.

## Supplementary Materials

The following are available online at https://www.mdpi.com/article/10.3390/ani11071947/s1, Table S1: Composition, analyzed and calculated nutrient composition of the experimental diets.

## References

[1] Moula N., Antoine-Moussiaux N., Decuypere E., Farnir F., Mertens K., de Baerdemaeker J., Leroy P. (2010). Comparative study of egg qualoty traits in two Belgian local breeds and two commercial lines of chicken. Arch. Geflügelk..

[2] Flock D.K., Schmutz M., Preisinger R. (2007). Optimierung der Eiqualität aus züchterischer Sicht. Züchtungskunde.

[3] Leenstra F., ten Napel J., Visscher J., van Sambeek F. (2016). Layer breeding programmes in changing production environments: A historic perspective. World’s Poult. Sci. J..

[4] Hernandez J.M. European Consumer Surveys about Egg Quality: How to Improve the Nutritional Value. Proceedings of the XIth European Symposium on the Quality of Eggs and Egg Products.

[5] Roberts J.R. (2004). Factors Affecting Egg Internal Quality and Egg Shell Quality in Laying Hens. J. Poult. Sci..

[6] Lordelo M., Cid J., Cordovil C.M.D.S., Alves S.P., Bessa R.J.B., Carolino I. (2020). A comparison between the quality of eggs from indigenous chicken breeds and that from commercial layers. Poult. Sci..

[7] Brade W., Flachowsky G., Schrader L. (2008). Legehuhnzucht und Eiererzeugung: Empfehlungen für die Praxis.

[8] Subcommittee on Poultry Nutrition, National Research Council (1994). Nutrient Requirements of Poultry.

[9] Rutkowski A., Hejdysz M., Kaczmarek S., Adamski M., Nowaczewski S., Jamroz D. (2017). The effect of addition of yellow lupin seeds (Lupinus luteus L.) to laying hen diets on performance and egg quality parameters. J. Anim. Feed Sci..

[10] Hernandez J.M., Beardsworth P., Weber G. (2001). Egg Quality—meeting consumer expectations. Int. Poult. Prod..

[11] Sauter E.A., Stadelman W.J., Carver J.S. (1952). Factors Affecting the Incidence of Blood Spots and Their Detection in Hen’s Eggs. Poult. Sci..

[12] Sirri F., Zampiga M., Soglia F., Meluzzi A., Cavani C., Petracci M. (2018). Quality characterization of eggs from Romagnola hens, an Italian local breed. Poult. Sci..

[13] Hocking P.M., Bain M., Channing C.E., Fleming R., Wilson S. (2003). Genetic variation for egg production, egg quality and bone strength in selected and traditional breeds of laying fowl. Br. Poult. Sci..

[14] Aydin R., Karaman M., Cicek T., Yardibi H. (2008). Black cumin (Nigella sativa L.) supplementation into the diet of the laying hen positively influences egg yield parameters, shell quality, and decreases egg cholesterol. Poult. Sci..

[15] Novak C., Scheideler S.E. (2001). Long-term effects of feeding flaxseed-based diets. 1. Egg production parameters, components, and eggshell quality in two strains of laying hens. Poult. Sci..

[16] Wilson P.B. (2017). Recent advances in avian egg science: A review. Poult. Sci..

[17] Muduuli D.S., Marquardt R.R., Guenter W. (1981). Effect of Dietary Vicine on the Productive Performance of Laying Chickens. Can. J. Anim. Sci..

[18] Lessire M., Gallo V., Prato M., Akide-Ndunge O., Mandili G., Marget P., Arese P., Duc G. (2017). Effects of faba beans with different concentrations of vicine and convicine on egg production, egg quality and red blood cells in laying hens. Animal.

[19] Gesellschaft für Ernährungsphysiologie (1999). Empfehlungen zur Energie-und Nährstoffversorgung der Legehennen und Masthühner (Broiler).

[20] Nolte T., Jansen S., Halle I., Scholz A.M., Simianer H., Sharifi A.R., Weigend S. (2020). Egg Production and Bone Stability of Local Chicken Breeds and Their Crosses Fed with Faba Beans. Animals.

[21] Li X.Y., Xu G.Y., Hou Z.C., Zhao R., Yang N. (2006). Variation of eggshell colour in different egg-type chickens. Arch. Geflügelk..

[22] Littell R.C. (2000). SAS System for Mixed Models.

[23] Rizzi C., Chiericato G.M. (2005). Organic farming production. Effect of age on the productive yield and egg quality of hens of two commercial hybrid lines and two local breeds. Ital. J. Anim. Sci..

[24] Olaboro G., Marquardt R.R., Campbell L.D., Fröhlich A.A. (1981). Purification, Identification and quantification of an Egg-weight-depressing factor (vicine) in fababeans (Vicia faba L.). J. Sci. Food Agric..

[25] Devaney J.A., Quisenberry J.H., Doran B.H., Bradley J.W. (1980). Dispersal of the northern fowl mite, Ornithonyssus sylviarum (Canestrini and Fanzago), and the chicken body louse, Menacanthus stramineus (Nitzsch), among thirty strains of egg-type hens in a caged laying house. Poult. Sci..

[26] Martínez J.A., Macarulla M.T., Marcos R., Larralde J. (1992). Nutritional outcome and immunocompetence in mice fed on a diet containing raw field beans (Vicia faba, var. minor) as the source of protein. Br. J. Nutr..

[27] Murillo A.C., Mullens B.A. (2017). A review of the biology, ecology, and control of the northern fowl mite, Ornithonyssus sylviarum (Acari: Macronyssidae). Vet. Parasitol..

[28] Fru-Nji F., Niess E., Pfeffer E. (2007). Effect of Graded Replacement of Soybean Meal by Faba Beans (Vicia faba L.) or Field Peas (Pisum sativum L.) in Rations for Laying Hens on Egg Production and Quality. J. Poult. Sci..

[29] Laudadio V., Tufarelli V. (2010). Treated fava bean (Vicia faba var. minor) as substitute for soybean meal in diet of early phase laying hens: Egg-laying performance and egg quality. Poult. Sci..

[30] Lewis C. (2010). The Illustrated Guide to Chickens: How to Choose Them—How to Keep Them.

[31] Pehle T., Hackstein Y. (2008). Dumonts Kleines Lexikon der Hühner: Aufzucht, Haltung, Rassen.

[32] Mori H., Takaya M., Nishimura K., Goto T. (2020). Breed and feed affect amino acid contents of egg yolk and eggshell color in chickens. Poult. Sci..

[33] Robblee A.R., Clandinin D.R., Hardin R.T., Milne G.R., Darlington K. (1977). STUDIES ON THE USE OF FABA BEANS IN RATIONS FOR LAYING HENS. Can. J. Anim. Sci..

[34] Dänner E.E. (2003). Einsatz von Vicin-/Convicin-armen Ackerbohnen (Vicia faba) bei Legehennen. Arch. Geflügelk..

[35] Réhault-Godbert S., Guyot N., Nys Y. (2019). The Golden Egg: Nutritional Value, Bioactivities, and Emerging Benefits for Human Health. Nutrients.

[36] Nolte T., Jansen S., Weigend S., Moerlein D., Halle I., Link W., Hummel J., Simianer H., Sharifi A.R. (2020). Growth Performance of Local Chicken Breeds, a High-Performance Genotype and Their Crosses Fed with Regional Faba Beans to Replace Soy. Animals.

