# Supplementary material for: Genotypic and Dietary Effects on Egg Quality of Local Chicken Breeds and Their Crosses Fed with Faba Beans
Source: Animals (Basel). 2021 Jun 29;11(7):1947. doi: 10.3390/ani11071947 (PMC8300114; doi:10.3390/ani11071947)
Supplement: Supplementary file 1 [file animals-11-01947-s001.zip › animals-1235427-supplementary.pdf]

Supplementary material

Table S1. Composition, analyzed and calculated nutrient composition of the experimental diets.

Item	Experiment A			Experiment B		
	Soy	VC+	VC-	Soy	VC+	VC-
			Ingredients (%)			
Wheat	40.39	29.78	29.78	40.39	29.78	29.78
Corn	10.00	10.89	10.89	10.00	10.89	10.89
Soybean meal (39.8% CP)	11.84	-	-	11.84	-	-
Blue sweet lupine cv. Boruta	21.00	21.13	21.13	21.00	21.13	21.13
Faba bean cv. Fuego	-	20.00	-	-	20.00	-
Faba bean cv. Tiffany	-	-	20.00	-	-	20.00
Soybean oil	4.00	5.77	5.77	4.00	5.77	5.77
Dicalcium phosphate	2.76	2.51	2.51	2.76	2.51	2.51
Calcium carbonate	8.39	8.25	8.25	8.39	8.25	8.25
Sodium chloride	0.32	0.25	0.25	0.32	0.25	0.25
DL-Methionine	0.21	0.28	0.28	0.21	0.28	0.28
Lysine	0.09	0.10	0.10	0.09	0.10	0.10
Tryptophan	-	0.04	0.04	-	0.04	0.04
Premix [1]	1.00	1.00	1.00	1.00	1.00	1.00
			Chemical composition			
Dry matter abs (%) [2]	89.60	89.50	89.50	90.90	91.20	90.90
Crude ash (g/kg DM) [2]	152.60	149.30	148.70	129.70	139.50	146.70
Crude protein (g/kg DM) [2]	182.40	171.90	185.30	185.20	202.10	184.10
Crude fat (g/kg DM) [2]	95.00	88.80	97.40	91.10	83.70	91.80
Crude fiber (g/kg DM) [2]	54.60	50.30	54.50	61.80	51.30	59.20
Starch (g/kg DM) [2]	362.60	393.10	349.50	365.90	349.5	347.00
Sucrose (g/kg DM) [2]	29.70	25.00	24.00	24.60	26.60	24.60
SFA (g/100g fat) [2]	17.70	17.00	16.10	17.6	16.80	16.40
MUFA (g/100g fat) [2]	22.50	22.60	22.80	22.70	22.80	21.80
PUFA (g/100g fat) [2]	59.80	60.40	61.10	59.6	60.40	61.70
Vicine (%) [2]	0.016	0.079	0.003	0.0	0.095	0.015
Convicine (%) [2]	0.006	0.037	0.002	0.0	0.039	0.004
VC (Vicin + Convicin; %) [3]	0.022	0.116	0.005	0.0	0.134	0.019
Tannin (mg/g) [2]	3.51	3.02	3.33	3.22	3.91	3.67
AMEn (MJ/kg) [3,4]	12.53	12.60	12.36	12.43	12.19	12.12
Methionine (%) [3]	0.42	0.44	0.44	0.42	0.44	0.44
Lysine (%) [3]	0.81	0.83	0.83	0.81	0.83	0.83
Tryptophan (%) [3]	0.16	0.17	0.17	0.16	0.17	0.17
Threonine (%) [3]	0.58	0.55	0.55	0.58	0.55	0.55

CP: crude protein, SFA: saturated fatty acids, MUFA: monounsaturated fatty acids, PUFA: polyunsaturated fatty acids, AME_n: nitrogen-corrected apparent metabolizable energy; [1] Premix – hens: feed additives (per kg premix): Vitamin A, 1,000,000 IU; Vitamin D3, 250,000 IU; Vitamin E, 2,000 mg; Vitamin B1, 250 mg; Vitamin B2, 700 mg; Vitamin B6, 400 mg; Vitamin B12, 2,000 µg; Vitamin K3, 400 mg; Nicotin amide, 4,000 mg; Calcium-D-pantothenate, 1,000 mg; Folic acid, 60 mg; Biotin, 2,500 µg; Choline chloride, 40,000 mg; Fe, 4,000 mg; Cu, 1,000 mg; Mn, 10,000 mg; Zn, 8,000 mg; I, 120 mg; Se, 25 mg; Co, 20.5 mg; Butylated hydroxy toluene (BHT), 12,500 mg; Beta-carotene, 400 mg; Canthaxanthin, 400 mg; [2] Analyzed; [3] Calculated; [4] Apparent metabolizable energy concentrations corrected to zero nitrogen balance (AMEn), calculated according to the energy estimation equation of the World's Poultry Association (Vogt, 1986)

Cited from: Nolte, T.; Jansen, S.; Halle, I.; Scholz, A. M.; Simianer, H.; Sharifi, A. R.; Weigend, S. Egg Production and Bone Stability of Local Chicken Breeds and Their Crosses Fed with Faba Beans. *Animals* **2020,** *10*. doi: 10.3390/ani10091480.
